# Predicting Academic Performance from Future-Oriented Daily Time Management Behavior: A LASSO-Based Study of First-Year College Students

**DOI:** 10.3390/bs15091242

**Published:** 2025-09-12

**Authors:** Mingzhang Zuo, Kunyu Wang, Pengxuan Tang, Meng Xiao, Xiaotang Zhou, Heng Luo

**Affiliations:** Faculty of Artificial Intelligence in Education, Central China Normal University, Wuhan 430079, China; mzzuo@mail.ccnu.edu.cn (M.Z.); pxtang@mails.ccnu.edu.cn (P.T.); xm678@mails.ccnu.edu.cn (M.X.); zhouxt2023@mails.ccnu.edu.cn (X.Z.)

**Keywords:** time-management, academic performance, first-year college students, predictive factors

## Abstract

This study examined how the time management behavior of first-year college students predicted their academic performance. Data on 44 objective indicators of daily time management behaviors were collected from 110 first-year students via a WeChat Mini Program, through one month of consecutive daily tracking. To identify stable predictors, Least Absolute Shrinkage and Selection Operator (LASSO) regression with 5000 bootstrap resamples was conducted, and variables with high selection frequency were subsequently entered Elastic Net regression to examine explanatory relationships. Six key behavioral indicators were found to predict overall academic performance. Subject-specific models revealed varying associations: time management behaviors appeared more influential in subjects such as Physical Education and English, while their role was less evident in Mathematics. The number and nature of retained predictors also differed across disciplines.

## 1. Introduction

First-year academic performance is a major concern for policymakers and universities (Fokkens-Bruinsma et al., 2021). Around 30% of students drop out due to academic disqualification, social anxiety, or emotional health issues (Aina et al., 2022; Craft, 2024; van Rooij et al., 2017). Academic disqualification, often resulting from failing to meet the 60% passing grade, can delay academic progress and affect mental health and future development (Lovin et al., 2022). This period also involves complex adjustments, requiring students to balance academic tasks, practical skills, social life, and personal well-being (Nelson, 2021; Wilcox et al., 2005). Shifting from a structured high school routine to autonomous time management is challenging (Schlossberg, 2011). Although individual differences in time management can affect learning outcomes, there is little formal training on the subject. And some attempts have been ineffective due to a lack of sufficiently accurate and actionable behavioral indicators (Islam et al., 2024).

Time management behaviors are multifaceted, typically examined through dimensions such as time planning, perceived time control, and time allocation. Britton and Tesser’s foundational work (Britton & Tesser, 1991) showed these behaviors explain 21% of the variance in cumulative college GPA, outperforming standardized test scores. Later studies confirmed this and highlighted key factors like planning and perceived time control (Adams & Blair, 2019). Effective time management also involves balancing activities through thoughtful time allocation. However, most research focuses on a single category of time management behaviors (Li et al., 2020), neglecting the interaction between multiple behaviors. To address this limitation, the present study conceptualizes time management behaviors as a multifaceted and recursive process that involves planning, monitoring, evaluating, and reflecting across diverse types of time-related activities, rather than focusing on any single behavioral category.

Current research suffers from data homogeneity and limited precision. Over half of studies rely on cross-sectional questionnaires, while long-term data collection is often confined to learning management platforms, with little input from time management tools (van Sluijs & Matzat, 2024). The accuracy and reliability of the data obtained are therefore questionable. Though direct evidence is lacking, the common practice of frequent scheduling among high achievers warrants attention (Alyami et al., 2021).

This study aimed to explore first-year college students’ daily time management behaviors and their impact on academic performance. We conducted an intensive two-month tracking of first-year university students’ time-management behavior, analyzing data from the final month of the semester alongside their first-semester academic performance. Least absolute shrinkage and selection operator (LASSO) regression was used to identify the behaviors that best predict academic performance. The main research questions are:

(1) How well do first-year college students manage their time and academic performance?
(2) Which daily time management behaviors predict students’ academic performance?

## 2. Literature Review

### 2.1. Academic Performance

Academic performance lacks a unified definition, often operationalized through metrics like grade point average or weighted average grade (Rodríguez-Hernández et al., 2020). In Chinese universities, weighted average grades (WAG, scored 0–100 and weighted by course credits) reflect students’ overall academic competency (Van Iddekinge et al., 2024). First-year academic performance significantly predicts later professional identity, confidence, and career outcomes (Colther et al., 2024).

Despite extensive research, predictors of first-year academic performance remain unclear. Cognitive and non-cognitive factors shape outcomes (Privado et al., 2024). Transitioning students face heightened non-curricular stressors (Waldeyer et al., 2022), requiring adaptation to campus life, social integration, and balancing academics with stress (Wrench et al., 2013). Resource constraints amplify these challenges (Malkoc & Tonietto, 2019), making effective time management for daily organization and leisure critical (Blagoev et al., 2024; Tonietto et al., 2021). Investigating time management as a non-cognitive factor may thus offer predictive insights into academic success (Kim et al., 2023).

### 2.2. Time Management Behavior as a Predictor of Academic Performance

The conceptualization of time management in academia is complex. It is often described as a multidimensional process personality trait, or behavior pattern that involves goal setting, prioritization, planning, time estimation, tracking, and deliberate time allocation (Macan et al., 1990).

#### 2.2.1. Phases of Time Management

According to self-regulated learning theory, time management in academic contexts can be understood through three interrelated stages (Wolters & Brady, 2021). The forethought phase centers on planning, where measurable and proximal goal-setting—such as defining task difficulty and focus—facilitates rational time allocation (Bird et al., 2024; Niedźwieńska et al., 2013). During the performance phase, monitoring time use (e.g., formal tracking or informal awareness of time passage) and evaluating adherence to planned schedules are critical for academic success (Dignath et al., 2023). In addition to duration, the frequency of time-related actions serves as an understudied yet key indicator of engagement quality (Solomon & Rothblum, 1984). Evaluation of time-related aspects of the task involves comparing the time devoted to the task with expected, planned, or external standards; for example, judging whether the task is being completed in the planned time (Kim et al., 2023). Self-assessment helps students reflect on progress, identify improvements, and adjust strategies for better time management (Yan et al., 2022).

The post-performance phase focuses on reflection. Students need to reflect on the time associated with completing the task, which triggers the process of attributing success or failure to the task (Kim et al., 2023). In turn, emotional responses and expectations of subsequent success may be influenced by their perceptions of time-related causes, such as the perception that the time invested in failing to complete a task is changeable and controllable, thus generating more positive emotions and expectations (Plant et al., 2005).

#### 2.2.2. Time Management Behavior Allocation

Time management for students encompasses both vertical processes (e.g., planning and scheduling) and horizontal time allocation (e.g., independent learning and extracurricular activities), which places emphasis on the number of activities carried out as well as the optimization of outcomes (Malkoc & Tonietto, 2019). There has been a noticeable trend in academic contexts toward a more circumscribed interpretation of time management that includes a predominant focus on the efficient use of time for learning activities (Xu, 2022). In addition to regular course learning and coursework, students must also carry out independent learning activities outside the classroom, such as professional practice and additional learning (Ersoy & Ayaz-Alkaya, 2024). College students’ extracurricular activities also go beyond leisure and sporting activities to include non-sporting pursuits such as reading, electronics, social, and hobbies (Foley et al., 2024; Fujiyama et al., 2021).

Building on the theoretical foundations reviewed above, this study conceptualizes time management behavior as a multidimensional construct encompassing four interrelated components: planning, monitoring, evaluating, and reflecting. These dimensions draw from models of self-regulated learning while also aligning with how students allocate their time across diverse academic and non-academic activities. Planning refers to the initial arrangement of study schedules prior to action, such as setting aside time for independent learning, or extracurricular participation. Monitoring involves students’ ongoing awareness and behavioral tracking during task execution, including the frequency and duration of activities. Evaluating captures students’ immediate post-task judgments of performance, whereas reflecting refers to end-of-day reviews of overall time use and learning outcomes. Informed by this conceptual framework, the present study further examines how these regulatory behaviors manifest across major categories of student activity, including course learning, social interaction, and leisure, among others. These dimensions and activity types jointly inform the development of the behavioral indicators used in the subsequent analysis, as detailed in the Section 3.

## 3. Methods

### 3.1. Participants and Context

The study was conducted at a research university in central China in the context of a compulsory, graded course titled “Freshman Orientation Seminar”, offered to all first-year students during their first semester. This course provides students with a systematic and comprehensive understanding of the characteristics and strategies of university study and seeks to build up a sense of time management and self-development gradually. As the course is not tied to a specific academic discipline and the data collected reflect students’ real-life time management behaviors, its design can be flexibly adapted to a wide range of undergraduate learning contexts. A total of 110 first-year college students between the ages of 17 and 20 from this course voluntarily participated in the research. These students were pursuing interdisciplinary majors related to computers. The sex distribution was 42.7% male and 57.3% female.

This study was conducted in accordance with the ethical standards of the Declaration of Helsinki. All participants were informed of the purpose and process of the study and signed an informed consent form before the study began. Participants could withdraw from the survey at any time if they felt uncomfortable. After the survey was completed, researchers gave participants a small reward to thank them for their continued participation.

### 3.2. Data Collection

#### 3.2.1. Daily Time-Management Behaviors

**Figure 1:**
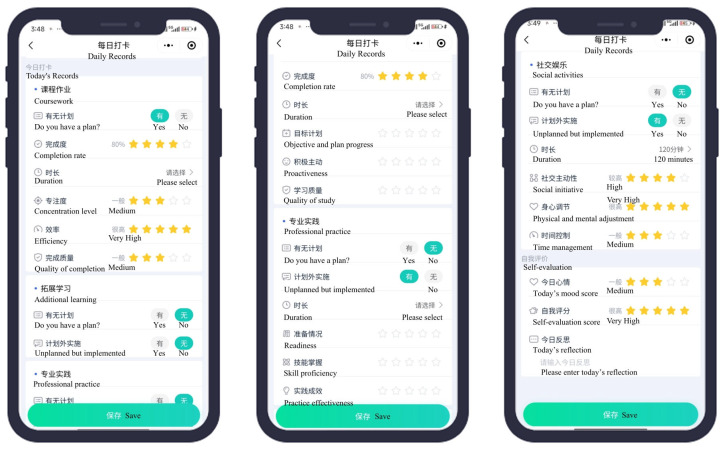
WeChat mini program: Self-Management Assistant (Higher-Education Edition).

To collect data on time-management behaviors, we developed a WeChat Mini Program titled Self-management Assistant (Higher-Education Edition). Existing commercial tools were either too fragmented, not tailored to academic contexts, or lacked the specific features required for our multi-dimensional behavioral tracking. Our Mini Program includes modules for planning daily activities, logging execution behaviors, and providing both immediate and end-of-day reflections (Appendix A.1; Figure 1).

Unlike conventional psychometric scales, the Mini Program functioned as a behavioral logging platform. To ensure usability, contextual fit, and system stability, we conducted a four-week internal pilot at the beginning of the study, during which participating students engaged in daily check-ins and activity logging. This phase served a dual purpose: its familiarized students with the program’s core functions and interface, and simultaneously allowed the research team to assess technical performance and collect user feedback. Based on students’ input, minor adjustments were made to the behavioral logging interface, notification settings, and activity categories to enhance clarity and usability. Following this phase, a four-week formal data collection period was conducted leading up to final examinations, a time when students typically experience increased academic pressure and complex scheduling demands. During this period, 110 first-year students contributed data across 44 daily time-management variables (Table 1), which were used for subsequent analysis. To align system-logged behaviors with theoretical constructs, we operationalized time management components based on distinct behavioral dimensions. For planning, only activities that were both planned and executed were retained, ensuring alignment with goal-directed enactment. Monitor was captured via logged activity durations and categorized task types. Evaluation included both task-specific self-assessments and daily overall ratings, while reflection focused on students’ end-of-day summaries. This structure preserved the conceptual distinction across components while accommodating the behavioral granularity captured by the Mini Program. All behavioral indicators were aggregated at the individual level, using either mean or total values depending on the variable type. All 44 behavioral indicators were derived from structured daily logs completed by participants through the Mini Program. Frequency variables (suffixes -A, -B, -D) represent the number of days in which a behavior was recorded, ranging from 0 to 28 days. Duration variables (suffix -C) reflect the average time spent per activity per day, recorded in minutes.

During the formal data collection phase, students were asked to complete daily behavioral logging for a period of 28 consecutive days. Participants who contributed fewer than 10% valid entries were excluded from analysis to ensure data reliability. To ensure data quality, we excluded participants whose number of missing daily entries exceeded 10% (i.e., fewer than 3 valid records). For the remaining participants, missing values were handled using mean imputation based on each student’s own available data. All 110 students included in the final analytic sample met the threshold for acceptable data completeness during the second (formal) data collection phase.

#### 3.2.2. Measurement of Academic Performance

Academic performance was assessed using final grades from all first-semester courses and five specific subjects (physical education, photography, Code, math, English), sourced directly from university records and calculated as weighted averages based on percentage-scale scores:

$$WAG=\frac{\sum_{i}\left(G_{i}\times C_{i}\right)}{\sum_{i}C_{i}}$$

where represents the grade for the *i*-th course on a 100-point scale, and is the weight of the course (based on the number of credits). The formula ensures that courses with higher weights (e.g., those with more credit) have a greater impact on the overall WAG.

These five subjects were selected based on both theoretical and practical considerations. Practically, while students may have had minor differences in their overall course schedules, the specific subjects whose grades were used in this study were compulsory for all first-year students. This ensured that the academic performance outcomes analyzed here were consistent and comparable across participants. Theoretically, they represent distinct domains of cognitive, behavioral, and affective demands. Physical Education emphasizes bodily regulation and consistent routines; Math and Code involve sustained analytical thinking and problem-solving; English requires cumulative language processing and frequent review; and Photography integrates both creativity and project-based execution. This diversity allowed us to investigate whether time-management behaviors show differentiated predictive effects depending on the type of learning task.

### 3.3. Data Analysis

All statistical analyses were performed using R (version 4.5.0), a flexible open-source environment for statistical computing.

#### 3.3.1. Descriptive Overview

Descriptive statistical analyses (including means, standard deviations, medians, and ranges) were conducted for all 44 time-management behavior variables to provide an overview of participants’ behavioral patterns. Given the number of variables, detailed results are presented in Appendix A for reference.

#### 3.3.2. Inferential Statistics

To assess the appropriateness of parametric assumptions, we conducted Shapiro–Wilk tests on all continuous variables. The results indicated that only Physical Education scores met the assumption of normality (*p* > 0.05), while all other variables significantly deviated from normality. Accordingly, we adopted non-parametric tests such as the Mann–Whitney U test and Spearman correlation for subsequent analyses.

#### 3.3.3. Bootstrap-LASSO Variable Selection and Elastic Net Predictive Modeling

To address the challenges of multicollinearity and high-dimensionality in the daily time-management behavior dataset (44 predictors with *n* = 110), we adopted a two-stage modeling strategy designed specifically for small-sample, high-dimensional settings. First, Least Absolute Shrinkage and Selection Operator (LASSO) regression (Tibshirani, 1996) was employed to perform variable selection through L1 regularization. Unlike traditional regression, LASSO imposes a penalty on the absolute size of coefficients, effectively shrinking less informative ones to zero, which not only mitigates overfitting but also enhances model interpretability in settings where the number of predictors may exceed the number of observations (*p* > n). Recent studies also indicate that penalized regression methods are well-suited for variable selection and model estimation, especially under small-sample and multicollinear conditions (Çıftçı et al., 2025).

To improve the robustness and replicability of our findings, we employed a two-stage analytical approach. In the first stage, we conducted 5000 bootstrap resamples combined with LASSO regression. In each iteration, the optimal regularization parameter (λ) was determined using 10-fold cross-validation via the cv.glmnet() function in R (Friedman et al., 2010). Variables with non-zero coefficients and selection frequency above 60% were retained (Bach, 2008; Meinshausen & Bühlmann, 2010). Their mean coefficients and average λ were calculated to reflect robust estimation across samples, thereby counteracting the variability introduced by sample noise.

In the second stage, the stable predictors identified through bootstrap-LASSO were entered an Elastic Net regression model, which integrates L1 (LASSO) and L2 (Ridge) penalties (Zou & Hastie, 2005). This hybrid regularization not only preserves the variable selection advantages of LASSO but also stabilizes coefficient estimation in the presence of collinearity. Importantly, Elastic Net has been shown to perform well in small-n, large-p contexts by balancing sparsity and grouping effects, making it particularly suitable for educational datasets where behavioral variables often exhibit intercorrelations. This final step enabled us to estimate more stable coefficients and better assess the predictive capacity of behavioral indicators for academic performance. Applied studies further indicate that Elastic Net, as an extension of LASSO, achieves stable performance in high-dimensional data contexts (Ehsanul Karim & Lei, 2025). Therefore, it was also used in this study to validate the robustness of the selection results.

## 4. Results

### 4.1. Q1: How Well Do First-Year College Students Manage Their Time and Perform Academically?

Students demonstrated generally good academic performance (overall WAG and course-specific grades above 70), though with significant variability. Math scores showed the lowest median and widest dispersion, potentially reflecting higher inherent difficulty, while the practical photography course achieved the highest and most consistent scores (Table 2). Notably, female students outperformed males in overall WAG, physical education (PE), and English, highlighting sex-based academic disparities.

Appendix A.2 presents descriptive statistics (mean, SD, median, min, max) for the 44 daily time management variables. Overall, students demonstrated notable variability in planning, duration, and evaluation across activity types. Among all activities, Electronic product use for entertainment (Epfe-C) showed the highest recorded average daily duration, reaching up to 529 minutes, while most academic and physical activities maintained much lower median durations (e.g., Physical exercise, PE-C: 13.5 minutes). In terms of behavioral frequency, Evaluation frequency for Classroom Learning (CL-D) and Professional Practices (PP-D) showed consistently high means (e.g., CL-D: M = 23.43, out of 28 possible days), indicating strong engagement in academic self-monitoring. Conversely, planning and implementation frequencies for physical and social activities (e.g., SA-A, PE-A) were low (means < 5), suggesting students seldom formally planned these types of activities. Large standard deviations in activities such as Epfe-C and SA-C indicate substantial inter-individual differences in leisure time allocation, with some students spending significantly more time than others. This diversity in behavioral engagement underscores the need to account for personalized time management strategies when analyzing academic outcomes.

**Figure 2:**
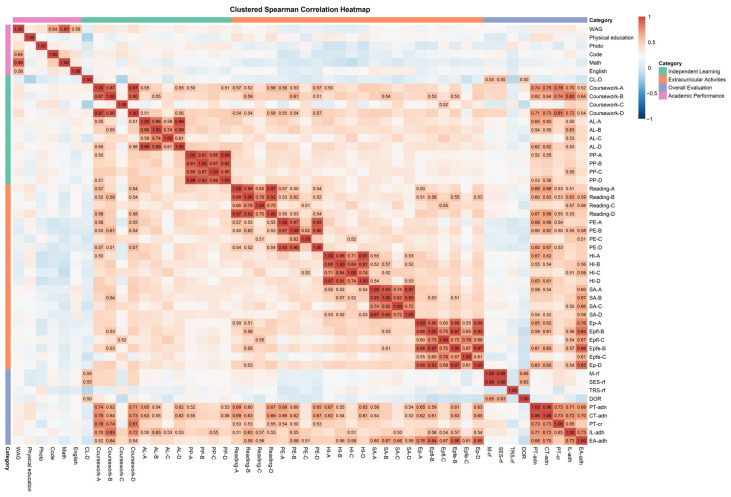
Matrix of bivariate partial correlations using Spearman rho coefficients. Note. WAG: Weighted average grade; A: planning and implementation frequency; B: hours recording frequency; C: average duration; D: evaluation frequency; Ep-A: planning and implementation frequency of Electronic products. CL: Classroom learning; AL: Additional learning; PP: Professional practices; PE: Physical exercise; HI: Hobbies and interests; SA: Social activities. Ep: Electronic products; Epfl: Electronics products for learning; Epfe: Electronics products for entertainment; M-rf: Recording frequency of mood scores; SES-rf: Recording frequency of self-evaluation scores; TRS-rf: Recording frequency of today’s reflection; DOR: Days of recording; PT-adn: Average daily number of planned tasks; CT-adn: Average daily number of completed tasks; PT-cr: Completion rate for planned tasks, IL-adh: Average daily hours of independent learning; EA-adh: Average daily hours of extracurricular activities.

Figure 2 the clustered heatmap presents Spearman correlations among all behavioral and outcome variables, grouped by four predefined categories: Independent Learning, Extracurricular Activities, Overall Evaluation, and Academic Performance. The red-to-blue color gradient indicates the direction and strength of correlation (red = positive, blue = negative, white = neutral), with numeric annotations displayed only for absolute correlation values exceeding 0.5 (|r| > 0.5). Clear clustering patterns are observed within each category, particularly in Extracurricular Activities and Overall Evaluation, suggesting internal consistency among related behaviors. Additionally, a set of moderate-to-strong positive correlations (r > 0.5) is found between overall evaluation indicators (e.g., completion rate, planned tasks) and academic performance (e.g., English, Math), implying that behavioral engagement metrics may serve as predictors of learning outcomes. Notably, Math showed a strong and consistent correlation with weighted average grades (r = 0.88), as well as a positive association with Code (r > 0.6), likely reflecting its academic weight and inherent difficulty.

### 4.2. Q2: Which Daily Time Management Behaviors Predict Academic Performance?

Table 3 presents the results of LASSO regression with 5000 bootstrap resamples for weighted average grade (WAG) and subject-specific grades. Variables with a selection frequency above 60% were retained for further modeling via Elastic Net regression, reflecting their relative stability and robustness across resampled datasets.

For the WAG, six behavioral indicators met the inclusion threshold. Notably, “Recording frequency of today’s reflection” (82.3%), “Coursework-average duration” (73.0%), and “Days of recording” (71.0%) emerged as the most stable predictors, with consistently positive coefficients. These results suggest that consistent self-monitoring and task execution behaviors are associated with improved general academic performance.

In contrast, predictors of subject-specific performance (e.g., PE, photo, coding, math, English) exhibited both commonalities and domain-specific patterns. “Coursework-average duration” and “Recording frequency of today’s reflection” were among the most frequently selected predictors across multiple subjects, indicating their general importance. However, domain-specific activities—such as “Physical exercise recording frequency” for PE, or “Additional learning-hours recording frequency” for photo—also contributed uniquely. Notably, certain predictors showed negative coefficients (e.g., “Professional practices-planning and implementation” in PE), suggesting that not all task-related engagement yields beneficial effects, potentially due to misalignment between effort and effectiveness. Overall, these findings highlight both the shared and distinct behavioral correlates of academic success across subjects.

Based on the bootstrap-enhanced LASSO analyses, a set of candidate predictors was identified for each outcome variable. To ensure robustness and interpretability, we applied a selection threshold whereby only variables with a bootstrap selection frequency equal to or exceeding 60% were retained for subsequent modeling. This threshold reflects a focus on model stability across resamples rather than strict statistical significance. Although some variables did not exhibit statistically significant 95% confidence intervals, their consistent re-selection across 5000 bootstrap samples indicates substantive predictive utility.

These retained predictors were then entered into an Elastic Net regression model (Table 4), which integrates both L1 (LASSO) and L2 (ridge) penalties. This approach allows for more reliable coefficient estimation, especially in the presence of correlated predictors, and helps mitigate overfitting in moderately sized datasets with high-dimensional features. The following section presents the results of the Elastic Net modeling based on the filtered predictors from the bootstrap LASSO stage.

In the Elastic Net model for overall weighted average grade (WAG), six predictors retained from the prior LASSO stage were included. Among them, coursework-evaluation frequency (β = 0.137) and coursework-average duration (β = 0.111) had the largest standardized coefficients, followed closely by days of recording (β = 0.105) and reflection frequency (β = 0.104). The penalty parameter was optimized at λ = 0.095 via cross-validation. The final model yielded an *R*^2^ of 0.204 and an adjusted *R*^2^ of 0.158, indicating moderate explanatory power. These results suggest that sustained coursework engagement and frequent reflection were particularly influential in predicting overall academic performance.

Across subject-specific models, the highest predictive accuracy was observed for Physical Education (PE) (adjusted *R*^2^= 0.255), which also retained the largest number of predictors. These included both positive indicators (e.g., *reflection frequency*, *task completion*, *recording behavior*) and negative ones (e.g., *reading-average duration*, *physical exercise-average duration*), reflecting a complex behavioral profile. English, in contrast, retained only four predictors, yet still achieved a relatively high adjusted R^2^ of 0.223, suggesting that a small set of key behavioral indicators may exert a strong influence on English performance. Mathematics had the lowest model fit (adjusted R^2^ = 0.095), indicating that other unmeasured factors may play a more prominent role in shaping math outcomes. Common predictors across models included *days of recording*, *coursework-average duration*, and *reflection frequency*, underscoring their cross-subject relevance. Meanwhile, subject-specific patterns emerged: for instance, *hobbies and interests* were negatively associated with outcomes in math and photo-related subjects, while *additional learning* contributed more prominently to English and photo performance. These findings highlight both the general and domain-specific behavioral dynamics underpinning academic success. Taken together, the most robust and generalizable behavioral predictors of academic success were consistency in data recording, sustained engagement with coursework, and reflective learning practices.

## 5. Discussion

This study used a 5000-iteration bootstrap LASSO regression to identify behavioral predictors of overall weighted average grade (WAG). Based on a selection frequency threshold of 60%, six variables were retained and included in a final Elastic Net model. Although the model explained only 15.8% of the variance in WAG, the aim was not to build a highly predictive model but to identify behavior patterns related to academic outcomes, particularly those involving time management and self-regulation. The explanatory power of the models varied across subject areas. While the mathematics model explained only 9.5% of the variance, models for English and Physical Education reached adjusted R^2^ values above 22%, suggesting subject-specific differences in how behavioral patterns relate to academic performance. Despite the modest variance explained, the selected predictors offer useful insights into students’ daily routines and may inform future research and interventions. The following discussion considers possible interpretations and implications of these findings.

### 5.1. Key Factors Predicting Academic Performance

#### 5.1.1. Days of Recording

Continuity of recording is crucial for mastering any skill. This necessitates a steadfast commitment, which is commonly referred to as perseverance (Duckworth et al., 2007). Studies have shown that individuals who possess passion and perseverance in working through challenges and adversity are more likely to achieve higher academic success compared to those who lack similar traits (Datu, 2021). Previous research has consistently shown that perseverance is a strong predictor of students’ academic performance (Thorsen et al., 2021), so it is not surprising that consistent recording of time management behaviors also predicts academic performance. As has been said before, students’ success in language acquisition and academic progress is largely influenced by their ability to persevere (Bi et al., 2024). Daily recording of time management behaviors strongly predicted English grades, showing the highest impact coefficient.

#### 5.1.2. Frequency Recording of Today’s Reflection

Frequent recording of today’s reflection helps students better understand their learning status and adjust strategies, benefiting performance across subjects (Lin et al., 2024). Theory of reflective practice emphasizes that students can improve their learning by reflecting on their learning process (Schon, 1984), as this enables individuals to identify and correct shortcomings in learning, which helps students to clarify their learning goals and develop a coherent learning program (Ames, 1992). Self-expectations vary among individuals, limiting the universality of self-evaluation scores (Yan et al., 2023). In contrast, reflection frequency is an objective predictor that raises students’ awareness of time use, helping them recognize procrastination and distractions. In this study, students evaluated both specific tasks and their overall daily performance. Such comprehensive reflection helps students focus on their overall progress rather than minor details (Shrivastava et al., 2024).

#### 5.1.3. Completion Rate for Planned Tasks

The completion rate for planned tasks, a key indicator of time management behavior, predicted not only overall grades but also performance in PE and English. While prior studies highlight the importance of planning (Janssen & Lazonder, 2024). This study shows that task completion is even more critical. Self-efficacy and mood improve when students see their plans well accomplished (Duckworth et al., 2019). This positive feedback can also lead students to show better time management competence and academic performance. By shaping study habits, motivation, and practical application, the completion rate predicted English and PE grades (Cui & Gardiner, 2025), both requiring sustained practice and repetition (Neumann et al., 2019).

#### 5.1.4. Average Duration and Evaluation Frequency of Coursework

The duration of the monitoring task has been the focus of most self-monitoring studies (Wheeler & Reis, 1991). Monitoring the time spent on tasks can increase accountability. Tracking their time often leads to more focused and intentional work. Monitoring the time students require to complete their coursework helps students avoid excessive breaks or distractions and improves their learning efficiency (Blake et al., 2020). Spending sufficient time on coursework and placing emphasis on evaluating the quality of completed work are both essential for promoting deeper understanding and academic improvement (Bernacki & Walkington, 2018). These are the important predictors for all courses, which are not particularly difficult but require sustained time investment. Spending enough time on coursework was associated with better academic performance, possibly by supporting students’ consolidation of key knowledge points.

#### 5.1.5. Time Management Behavior Regarding Electronics

Electronics play an important role in the daily life of college students. The issue of electronic device use has also been identified as an important factor affecting students’ time management (Balta et al., 2020). Many studies have shown that increased time spent using electronic devices can lead to dependency and negatively affect academic performance (Saunders & Vallance, 2017). However, contrary to some previous studies, we found that time management behaviors in the use of electronic devices did not significantly predict academic performance. Some students can manage their screen time systematically and effectively to ensure that it does not interfere with their studies—indeed, it can even contribute to their academic success (Moretta et al., 2022). There was no significant association between time management behaviors with electronic devices and academic performance, and this discrepancy between use and addiction may be the reason for this. Our data collection from student self-reports may also not reflect the actual situation, although we took steps to encourage students to report on their daily time management behaviors truthfully.

### 5.2. Subject-Specific Differences in Predictive Model

Some notable differences appeared to emerge among the Physical Education, English, and Mathematics models in terms of both the number and nature of the predictive behavioral indicators. Among the subject-specific models, Physical Education and English demonstrated relatively high levels of explanatory power. However, the composition of their predictive structures differed considerably. These differences may indicate that certain subjects tend to be better explained by a wide behavioral profile, while others may rely more on a focused set of self-regulatory habits.

The Physical Education model retained a broader set of behavioral indicators, encompassing both positive and negative predictors. This diversity may reflect the multifaceted nature of physical education, which involves not only cognitive and academic engagement but also physical activity, self-discipline, and lifestyle routines (Shen et al., 2007). The English model included fewer predictors but still achieved comparable explanatory power. The retained behaviors, such as task completion and daily reflection, may reflect focused aspects of academic persistence and language development. This aligns with prior research indicating that language learning is supported by process-oriented and self-regulated behaviors (Oxford, 2016; Panadero, 2017).

In contrast, the Math model retained the lowest level of explanatory power, suggesting a relatively limited, though still observable, behavioral association. This is in line with earlier findings indicating that time management and behavioral tracking may have limited predictive value for mathematics achievement (Ganzon & Edig, 2022). Mathematics performance may depend more on conceptual understanding and domain-specific reasoning, which are less easily captured through time-based self-tracking (Rittle-Johnson & Schneider, 2015). While some studies have reported stronger associations using behavioral data (Romero et al., 2024), the comparatively weaker predictability observed here highlights the potential importance of considering subject-specific mechanisms. These preliminary findings point to the value of tailoring behavioral models to disciplinary contexts.

### 5.3. Implications

This exploratory study provides preliminary insights into the potential associations between specific time management behaviors and academic performance among first-year college students. Although the overall explanatory power of the model is limited, this may be expected given the multifactorial nature of academic achievement and the study’s emphasis on a focused set of behavioral indicators. Nevertheless, the behaviors identified, such as planning and reflection, are concrete and modifiable, which may render them valuable targets for intervention despite their modest predictive utility. Rather than examining overall time use, future research may benefit from unpacking the contributions of distinct processes, such as planning, time monitoring, behavioral tracking, and reflective evaluation. It may also be useful to explore how different categories of activities relate to academic outcomes across subject areas. Students could benefit from becoming more aware of their time-use patterns and gradually developing more adaptive habits, such as consistently recording their learning behaviors and adjusting time allocation in response to specific academic demands (e.g., Nicol, 2021). While these findings should be interpreted with caution, they may inform tentative instructional strategies. For example, instructors may support students’ reflective habits by embedding self-reflection prompts into assignments, encouraging the use of digital journals or weekly feedback summaries, and modeling metacognitive questioning in class discussions. Institutions might also integrate lightweight reflection tools into learning management systems to normalize the practice of periodic self-monitoring. Educators might encourage students to develop individualized study routines or incorporate time-awareness and reflection elements into course design (e.g., Plass & Pawar, 2020). Given the sample and model constraints, however, further validation with larger and more diverse populations is warranted before drawing firm conclusions or implementing broader pedagogical applications.

### 5.4. Limitations and Future Research

This study has several limitations. First, as an exploratory study, the relatively small sample size (*n* = 110) compared to the number of predictors (44) remains a key limitation. While we applied 5000 bootstrap resamples, LASSO-based selection, and Elastic Net regression to enhance model stability, these methods cannot fully offset the risks associated with limited data. Future research with larger and more diverse samples is needed to replicate and extend these findings. Second, although behavioral data were recorded daily, all variables were aggregated at the student level for modeling. This design captures between-student differences but overlooks within-student variability. Future research may consider mixed-effects models to better account for nested data structures. Third, most frequency-based behavioral indicators were automatically recorded by the system, whereas some duration-related variables required self-input and may have been affected by recall bias. With careful attention to the ethical issues surrounding sensor-based tracking, future research could combine objective data sources with validated self-report measures to enhance both measurement accuracy and psychometric quality. Finally, the predictors used in this study were limited to time management behaviors captured by the current system (under 50% variance explained). While these offer valuable insights into self-regulated learning, their explanatory power for academic performance is limited. Future research should incorporate additional cognitive, motivational, and contextual variables, and explore subject-specific predictors to improve model accuracy. In addition, dimensionality reduction techniques such as principal component analysis (PCA) may be explored in future studies to address multicollinearity more structurally, particularly in contexts where model interpretability is not the primary concern.

## 6. Conclusions

This study offers preliminary evidence for associations between specific time management behaviors and academic performance in first-year university students. It provides a novel perspective that explains the ranking of the importance of planning, monitoring, and evaluation in time management research. We found that the duration of special categories of activities and adherence to reflective habits were also important factors affecting academic performance. Predictors of time management behaviors varied across disciplines. The importance of time management behavior was more prominent for Physical Education and English learning, but performance in math courses was less affected but could be improved by increasing engagement in coursework and professional practices. While these findings offer valuable insights, it is important to note that time management represents only one of many factors influencing academic performance. Other cognitive, motivational, or contextual variables, such as subject-specific aptitude, interest, or workload, may also play substantial roles. Considering the observed time management behavioral predictors, educators and practitioners may tentatively encourage first-year students to not only engage in goal planning but also develop habits of regular self-reflection, which emerged as a potentially impactful behavior in relation to academic performance.

## Figures and Tables

**Table 1:** Digital records of daily time management behaviors.

Category	Variable	Indicators *
Independent Learning	Coursework	A\B\C\D
	Additional learning	A\B\C\D
	Professional practices	A\B\C\D
	Classroom learning	D
Extracurricular Activities	Reading	A\B\C\D
	Physical exercise	A\B\C\D
	Hobbies and interests	A\B\C\D
	Social activities	A\B\C\D
	Electronic products	Ep-A, Epfl-B, Epfl-C, Epfe-B, Epfe-C, Ep-D
Overall Evaluation	Recording frequency of mood/today’s reflection/self-evaluation scores Days of recording Average daily number of planned tasks Average daily number of completed tasks Completion rate for planned tasks Average daily hours of independent learning Average daily hours of extracurricular activities	

* Note. A: planning and implementation frequency; B: hours recording frequency; C: average duration; D: evaluation frequency. Ep: Electronic products; Epfl: Electronics products for learning; Epfe: Electronics products for entertainment; Ep-A: planning and implementation frequency of Electronic products.

**Table 2:** Demographic differences in academic performance.

Grade	Median	IQR	Sex	Median	IQR	U	P	RBC
WAG	80.33	9.35	Male	78.17	11.12	1032	0.007	0.258
			Female	82.30	9.82			
Physical Education	74.85	15.50	Male	70.00	12.50	1010.5	0.005	0.271
			Female	79.00	14.00			
Photography	83.39	4.57	Male	83.00	3.60	1301	0.279	0.103
			Female	83.50	5.55			
Code	76.65	11.70	Male	74.50	10.60	1176	0.066	0.175
			Female	79.50	13.00			
Math	73.40	20.35	Male	72.60	19.50	1452	0.866	0.016
			Female	73.60	20.70			
English	78.80	11.73	Male	75.20	13.00	1007.5	0.004	0.273
			Female	81.00	8.30			

Note. IQR: Interquartile Range. RBC: rank-biserial correlation.

**Table 3:** Stable predictors identified by bootstrap LASSO for academic performance.

Grade	*λ*	Variable	Freq	Coef (SE)
WAG	0.05740591	Recording frequency of today’s reflection	82.30%	0.1 (0.08) *
		Coursework—average duration	73.00%	0.11 (0.12)
		Days of recording	71.00%	0.2 (0.23) *
		Completion rate for planned tasks	67.92%	0.14 (0.18)
		Coursework—evaluation frequency	63.96%	0.16 (0.36) *
		Professional practices-average duration	63.20%	0.03 (0.08)
PE	0.037899701	Recording frequency of today’s reflection	96.14%	0.23 (0.12) *
		Completion rate for planned tasks	78.76%	0.16 (0.16)
		Reading—planning and implementation frequency	74.58%	0.13 (0.18) *
		Classroom learning—evaluation frequency	69.06%	−0.12 (0.14)
		Days of recording	68.70%	0.17 (0.19) *
		Professional practices-planning and implementation frequency	67.24%	−0.22 (0.33)
		Average daily number of planned tasks	66.44%	0.29 (0.33) *
		Physical exercise—hours recording frequency	64.20%	0.14 (0.2) *
		Electronics products for entertainment-average duration	63.68%	0.05 (0.1)
		Electronics products for learning—average duration	63.54%	0.07 (0.11)
		Reading—average duration	62.68%	−0.07 (0.12)
		Physical exercise—average duration	62.56%	−0.04 (0.1)
		Additional learning—average duration	61.26%	0.03 (0.11)
		Average daily hours of independent learning	61.24%	−0.15 (0.21)
		Coursework-average duration	60.26%	0.03 (0.1)
photo	0.052164846	Recording frequency of today’s reflection	98.24%	0.22 (0.1) *
		Coursework—average duration	89.44%	0.16 (0.12) *
		Additional learning—hours recording frequency	72.36%	0.12 (0.14) *
		Hobbies and interests—hours recording frequency	64.14%	−0.09 (0.11)
		Professional practices—average duration	62.60%	0.03 (0.09)
		Additional learning—average duration	61.68%	0.04 (0.07)
code	0.058424878	Coursework—average duration	87.40%	0.17 (0.12) *
		Professional practices—average duration	78.52%	0.08 (0.09) *
		Days of recording	71.40%	0.14 (0.16) *
		Additional learning—average duration	64.34%	0.07 (0.1)
math	0.070031437	Coursework—evaluation frequency	70.14%	0.25 (0.48) *
		Hobbies and interests—average duration	68.82%	−0.17 (0.17)
		Professional practices—average duration	63.32%	0.05 (0.09)
English	0.058617444	Days of recording	93.82%	0.42 (0.29) *
		Coursework—average duration	77.28%	0.12 (0.13) *
		Additional learning—average duration	71.40%	0.08 (0.1) *
		Completion rate for planned tasks	70.68%	0.15 (0.18) *

Note. WAG: Weighted average grade; PE: Physical education; *λ* = the average bootstrapped *λ*; Coef (SE) = the average bootstrapped coefficient and its standard error; Freq = selection frequency across 5000 bootstrap LASSO iterations; * indicates 95% CI excludes zero (statistically significant).

**Table 4:** Elastic Net regression model predicting overall and subject-specific grades.

	WAG	PE	Photo	Code	Math	English
Coursework-evaluation frequency	**0.137**				**0.243**	
Coursework—average duration	0.111	0.083	0.179	**0.217**		0.167
Days of recording	0.105	0.187		0.160		**0.375**
Recording frequency of today’s reflection	0.104	0.268	**0.259**			
Completion rate for planned tasks	0.096	0.159				0.154
Professional practices—average duration	0.063		0.108	0.113	0.169	
Additional learning—hours recording frequency			0.196			
Average daily number of planned tasks		**0.325**				
Physical exercise—hours recording frequency		0.165				
Reading—planning and implementation frequency		0.156				
Electronics products for learning—average duration		0.091				
Electronics products for entertainment—average duration		0.049				
Additional learning—average duration		0.018	0.050	0.088		0.089
Physical exercise—average duration		(0.064)				
Reading—average duration		(0.090)				
Classroom learning—evaluation frequency		(0.170)				
Professional practices—planning and implementation frequency		(0.259)				
Average daily hours of independent learning		(0.312)				
Hobbies and interests—hours recording frequency			(0.171)			
Hobbies and interests—average duration					(0.195)	
*λ*	0.095	0.018	0.029	0.014	0.010	0.023
R^2^	0.204	0.357	0.263	0.147	0.120	0.251
R^2^_adj_	0.158	0.255	0.220	0.115	0.095	0.223

Note. WAG: Weighted average grade; PE: Physical education. Bold values indicate the strongest predictor (largest coefficient). Negative values are shown in parentheses (red color).

## Data Availability

The data used to support the findings of this study are available at Mendeley Data (https://data.mendeley.com/preview/psspn8h5dg?a=93d7b70f-2e85-4e51-a189-931cce08b70c, accessed on 10 September 2025).

## Appendix A

### Appendix A.2. Descriptive Statistics for 44 Daily Time Management Variables

**Variable**	**Mean**	**SD**	**Median**	**Min**	**Max**	**Variable**	**Mean**	**SD**	**Median**	**Min**	**Max**
*CL-D*	23.43	5.92	27	2	28	*HI-B*	2.73	4.87	0	0	25
*Coursework-A*	14.85	9.81	17.5	0	28	*HI-C*	35.53	54.74	0	0	240
*Coursework-B*	13.23	9.94	13	0	28	*HI-D*	3.05	5.11	1	0	27
*Coursework-C*	85.05	70.70	94.87	0	357.69	*SA-A*	3.26	4.84	2	0	27
*Coursework-D*	14.29	9.92	15.5	0	28	*SA-B*	3.20	4.62	1	0	21
*AL-A*	4.52	5.95	2	0	27	*SA-C*	58.97	89.51	7.1	0	420
*AL-B*	3.95	5.48	1	0	26	*SA-D*	3.35	4.85	1.5	0	27
*AL-C*	42.85	50.92	12	0	210	*Ep-A*	8.35	10.09	3	0	28
*AL-D*	4.37	5.79	2	0	27	*Epfl-B*	7.62	9.47	2	0	28
*PP-A*	1.79	3.97	0	0	27	*Epfl-C*	58.62	74.68	18.97	0	387.27
*PP-B*	1.85	4.31	0	0	27	*Epfe-B*	8.15	10.07	2	0	28
*PP-C*	26.06	51.67	0	0	260	*Epfe-C*	84.96	101.66	30.165	0	529.09
*PP-D*	1.79	3.98	0	0	27	*Ep-D*	8.28	9.85	2.5	0	28
*Reading-A*	5.87	7.84	2	0	28	*M-rf*	23.26	7.56	27	0	28
*Reading-B*	5.25	7.60	1.5	0	28	*SES-rf*	23.18	7.67	27	0	28
*Reading-C*	34.66	45.22	11.6	0	197.84	*TRS-rf*	3.90	7.43	0	0	28
*Reading-D*	5.75	7.77	2	0	28	*DOR*	25.92	4.55	28	3	28
*PE-A*	3.95	5.54	2	0	28	*PT-adn*	2.34	1.85	2.015	0	11.54
*PE-B*	3.89	5.76	1	0	28	*CT-adn*	2.40	1.84	2.36	0	11.54
*PE-C*	38.05	50.86	13.5	0	360	*PT-cr*	0.61	0.32	0.71	0	1
*PE-D*	4.46	6.76	2	0	28	*IL-adh*	120.03	105.17	101.25	0	582
*HI-A*	3.01	5.09	1	0	27	*EA-adh*	120.24	143.13	60.59	0	708
Note. A: planning and implementation frequency; B: hours recording frequency; C: average duration; D: evaluation frequency; Ep-A: planning and implementation frequency of Electronic products. CL: Classroom learning; AL: Additional learning; PP: Professional practices; PE: Physical exercise; HI: Hobbies and interests; SA: Social activities. Ep: Electronic products; Epfl: Electronics products for learning; Epfe: Electronics products for entertainment; M-rf: Recording frequency of mood scores; SES-rf: Recording frequency of self-evaluation scores; TRS-rf: Recording frequency of today’s reflection; DOR: Days of recording; PT-adn: Average daily number of planned tasks; CT-adn: Average daily number of completed tasks; PT-cr: Completion rate for planned tasks, IL-adh: Average daily hours of independent learning; EA-adh: Average daily hours of extracurricular activities.											

## References

[B1-behavsci-15-01242] Adams R. V., Blair E. (2019). Impact of time management behaviors on undergraduate engineering students’ performance. Sage Open.

[B2-behavsci-15-01242] Aina C., Baici E., Casalone G., Pastore F. (2022). The determinants of university dropout: A review of the socio-economic literature. Socio-Economic Planning Sciences.

[B3-behavsci-15-01242] Alyami A., Abdulwahed A., Azhar A., Binsaddik A., Bafaraj S. M. (2021). Impact of time-management on the student’s academic performance: A cross-sectional study. Creative Education.

[B4-behavsci-15-01242] Ames C. (1992). Classrooms: Goals, structures, and student motivation. Journal of Educational Psychology.

[B5-behavsci-15-01242] Bach F. R. (2008). Bolasso: Model consistent Lasso estimation through the bootstrap. Proceedings of the 25th international conference on machine learning (ICML’08).

[B6-behavsci-15-01242] Balta S., Emirtekin E., Kircaburun K., Griffiths M. D. (2020). Neuroticism, trait fear of missing out, and phubbing: The mediating role of state fear of missing out and problematic Instagram use. International Journal of Mental Health and Addiction.

[B7-behavsci-15-01242] Bernacki M. L., Walkington C. (2018). The role of situational interest in personalized learning. Journal of Educational Psychology.

[B8-behavsci-15-01242] Bi J., Izadpanah S., Mohammadi Z., Rezaei Y. M. (2024). Investigating the impact of technology-based education on academic motivation, academic perseverance, and academic self-efficacy in English language learning skills. Education and Information Technologies.

[B9-behavsci-15-01242] Bird M. D., Swann C., Jackman P. C. (2024). The what, why, and how of goal setting: A review of the goal-setting process in applied sport psychology practice. Journal of Applied Sport Psychology.

[B10-behavsci-15-01242] Blagoev B., Hernes T., Kunisch S., Schultz M. (2024). Time as a research lens: A conceptual review and research agenda. Journal of Management.

[B11-behavsci-15-01242] Blake R. J., Guillén G., Thorne S. L. (2020). Brave new digital classroom: Technology and foreign language learning.

[B12-behavsci-15-01242] Britton B. K., Tesser A. (1991). Effects of time-management practices on college grades. Journal of Educational Psychology.

[B15-behavsci-15-01242] Colther C., Espinoza O., Sandoval L., McGinn N. (2024). Impact of university academic performance on financial returns to education in Chile. International Journal of Educational Research.

[B13-behavsci-15-01242] Craft S. (2024). College dropout rates.

[B16-behavsci-15-01242] Cui X., Gardiner I. A. (2025). Investigating students’ academic language-related challenges and their interplay with English proficiency and self-efficacy on EMI success in Transnational Education (TNE) programs in China. English for Specific Purposes.

[B14-behavsci-15-01242] Çıftçı A. T., Yildirim D. D., Sucu D. H. (2025). A comparison of penalized regression methods on model estimation and variable selection: A simulation study. Turkiye Klinikleri Journal of Biostatistics.

[B17-behavsci-15-01242] Datu J. A. D. (2021). Beyond passion and perseverance: Review and future research initiatives on the science of grit. Frontiers in Psychology.

[B18-behavsci-15-01242] Dignath C., van Ewijk R., Perels F., Fabriz S. (2023). Let learners monitor the learning content and their learning behavior! A meta-analysis on the effectiveness of tools to foster monitoring. Educational Psychology Review.

[B19-behavsci-15-01242] Duckworth A. L., Peterson C., Matthews M. D., Kelly D. R. (2007). Grit: Perseverance and passion for long-term goals. Journal of Personality and Social Psychology.

[B20-behavsci-15-01242] Duckworth A. L., Taxer J. L., Eskreis-Winkler L., Galla B. M., Gross J. J. (2019). Self-control and academic achievement. Annual Review of Psychology.

[B21-behavsci-15-01242] Ehsanul Karim M., Lei Y. (2025). Is there a competitive advantage to using multivariate statistical or machine learning methods over the Bross formula in the hdPS framework for bias and variance estimation?. PLoS ONE.

[B22-behavsci-15-01242] Ersoy E., Ayaz-Alkaya S. (2024). Academic self-efficacy, personal responsibility, and readiness for professional practice in nursing students: A descriptive and correlational design. Nurse Education Today.

[B23-behavsci-15-01242] Fokkens-Bruinsma M., Vermue C., Deinum J.-F., Van Rooij E. (2021). First-year academic achievement: The role of academic self-efficacy, self-regulated learning and beyond classroom engagement. Assessment & Evaluation in Higher Education.

[B24-behavsci-15-01242] Foley C., Darcy S., Hergesell A., Almond B., McDonald M., Brett E. (2024). University-based sport and social clubs and their contribution to the development of graduate attributes. Active Learning in Higher Education.

[B25-behavsci-15-01242] Friedman J. H., Hastie T., Tibshirani R. (2010). Regularization paths for generalized linear models via coordinate descent. Journal of Statistical Software.

[B26-behavsci-15-01242] Fujiyama H., Kamo Y., Schafer M. (2021). Peer effects of friend and extracurricular activity networks on students’ academic performance. Social Science Research.

[B27-behavsci-15-01242] Ganzon W. J., Edig M. M. (2022). Time management and self-directed learning as predictors of academic performance of students in mathematics. Journal of Social, Humanity, and Education.

[B28-behavsci-15-01242] Islam A., Kwon S., Masood E., Prakash N., Sabarwal S., Saraswat D. (2024). All pain and no gain: When goal setting leads to more effort but no gains in test scores. Economics of Education Review.

[B29-behavsci-15-01242] Janssen N., Lazonder A. W. (2024). Meta-analysis of interventions for monitoring accuracy in problem solving. Educational Psychology Review.

[B30-behavsci-15-01242] Kim Y., Yu S. L., Wolters C. A., Anderman E. M. (2023). Self-regulatory processes within and between diverse goals: The multiple goals regulation framework. Educational Psychologist.

[B31-behavsci-15-01242] Li L., Gao H., Xu Y. (2020). The mediating and buffering effect of academic self-efficacy on the relationship between smartphone addiction and academic procrastination. Computers & Education.

[B32-behavsci-15-01242] Lin C.-C., Cheng E. S. J., Huang A. Y. Q., Yang S. J. H. (2024). DNA of learning behaviors: A novel approach of learning performance prediction by NLP. Computers and Education: Artificial Intelligence.

[B33-behavsci-15-01242] Lovin D., Bernardeau-Moreau D. (2022). Stress among students and difficulty with time management: A study at the university of Galați in Romania. Social Sciences.

[B34-behavsci-15-01242] Macan T. H., Shahani C., Dipboye R. L., Phillips A. P. (1990). College students’ time management: Correlations with academic performance and stress. Journal of Educational Psychology.

[B35-behavsci-15-01242] Malkoc S. A., Tonietto G. N. (2019). Activity versus outcome maximization in time management. Current Opinion in Psychology.

[B36-behavsci-15-01242] Meinshausen N., Bühlmann P. (2010). Stability selection. Journal of the Royal Statistical Society: Series B (Statistical Methodology).

[B37-behavsci-15-01242] Moretta T., Buodo G., Demetrovics Z., Potenza M. N. (2022). Tracing 20 years of research on problematic use of the internet and social media: Theoretical models, assessment tools, and an agenda for future work. Comprehensive Psychiatry.

[B38-behavsci-15-01242] Nelson L. J. (2021). The theory of emerging adulthood 20 years later: A look at where it has taken us, what we know now, and where we need to go. Emerging Adulthood.

[B39-behavsci-15-01242] Neumann H., Padden N., McDonough K. (2019). Beyond English language proficiency scores: Understanding the academic performance of international undergraduate students during the first year of study. Higher Education Research & Development.

[B40-behavsci-15-01242] Nicol D. (2021). The power of internal feedback: Exploiting natural comparison processes. Assessment & Evaluation in Higher Education.

[B41-behavsci-15-01242] Niedźwieńska A., Janik B., Jarczyńska A. (2013). Age-related differences in everyday prospective memory tasks: The role of planning and personal importance. International Journal of Psychology.

[B42-behavsci-15-01242] Oxford R. L. (2016). Teaching and researching language learning strategies: Self-regulation in context.

[B43-behavsci-15-01242] Panadero E. (2017). A review of self-regulated learning: Six models and four directions for research. Frontiers in Psychology.

[B44-behavsci-15-01242] Plant E. A., Ericsson K. A., Hill L., Asberg K. (2005). Why study time does not predict grade point average across college students: Implications of deliberate practice for academic performance. Contemporary Educational Psychology.

[B45-behavsci-15-01242] Plass J. L., Pawar S., Plass J. L., Mayer R. E., Homer B. D. (2020). Adaptivity and personalization in games for learning. Handbook of game-based learning.

[B46-behavsci-15-01242] Privado J., Pérez-Eizaguirre M., Martínez-Rodríguez M., Ponce-de-León L. (2024). Cognitive and non-cognitive factors as predictors of academic performance. Learning and Individual Differences.

[B47-behavsci-15-01242] Rittle-Johnson B., Schneider M., Cohen Kadosh R., Dowker A. (2015). Developing conceptual and procedural knowledge of mathematics. The Oxford handbook of numerical cognition.

[B48-behavsci-15-01242] Rodríguez-Hernández C. F., Cascallar E., Kyndt E. (2020). Socio-economic status and academic performance in higher education: A systematic review. Educational Research Review.

[B49-behavsci-15-01242] Romero M., Casadevante C., Santacreu J. (2024). Time management, fluid intelligence and academic achievement. Psychological Studies.

[B50-behavsci-15-01242] Saunders T. J., Vallance J. K. (2017). Screen time and health indicators among children and youth: Current evidence, limitations and future directions. Applied Health Economics and Health Policy.

[B51-behavsci-15-01242] Schlossberg N. (2011). Happiness relates to whether you are «Off-Time», «On-Time», «Out of Time». Psychology Today.

[B52-behavsci-15-01242] Schon D. A. (1984). The reflective practitioner: How professionals think in action.

[B53-behavsci-15-01242] Shen B., McCaughtry N., Martin J. (2007). The influence of self-determination in physical education on leisure-time physical activity behavior. Research Quarterly for Exercise and Sport.

[B54-behavsci-15-01242] Shrivastava A., Azhar H., Hyland L. (2024). A personal journey of studying positive psychology: Reflections of undergraduate students in the United Arab Emirates. Teaching of Psychology.

[B55-behavsci-15-01242] Solomon L. J., Rothblum E. D. (1984). Academic procrastination: Frequency and cognitive-behavioral correlates. Journal of Counseling Psychology.

[B56-behavsci-15-01242] Thorsen C., Yang Hansen K., Johansson S. (2021). The mechanisms of interest and perseverance in predicting achievement among academically resilient and non-resilient students: Evidence from Swedish longitudinal data. British Journal of Educational Psychology.

[B57-behavsci-15-01242] Tibshirani R. (1996). Regression shrinkage and selection via the lasso. Journal of the Royal Statistical Society: Series B (Methodological).

[B58-behavsci-15-01242] Tonietto G. N., Malkoc S. A., Reczek R. W., Norton M. I. (2021). Viewing leisure as wasteful undermines enjoyment. Journal of Experimental Social Psychology.

[B59-behavsci-15-01242] Van Iddekinge C. H., Arnold J. D., Krivacek S. J., Frieder R. E., Roth P. L. (2024). Making the grade? A meta-analysis of academic performance as a predictor of work performance and turnover. The Journal of Applied Psychology.

[B60-behavsci-15-01242] van Rooij E. C. M., Jansen E. P. W. A., van de Grift W. J. C. M. (2017). First-year university students’ academic success: The importance of academic adjustment. European Journal of Psychology of Education.

[B61-behavsci-15-01242] van Sluijs M., Matzat U. (2024). Predicting time-management skills from learning analytics. Journal of Computer Assisted Learning.

[B62-behavsci-15-01242] Waldeyer J., Dicke T., Fleischer J., Guo J., Trentepohl S., Wirth J., Leutner D. (2022). A moderated mediation analysis of conscientiousness, time management strategies, effort regulation strategies, and university students’ performance. Learning and Individual Differences.

[B63-behavsci-15-01242] Wheeler L., Reis H. T. (1991). Self-recording of everyday life events: Origins, types, and uses. Journal of Personality.

[B64-behavsci-15-01242] Wilcox P., Winn S., Fyvie-Gauld M. (2005). It was nothing to do with the university, it was just the people: The role of social support in the first-year experience of higher education. Studies in Higher Education.

[B65-behavsci-15-01242] Wolters C. A., Brady A. C. (2021). College students’ time management: A self-regulated learning perspective. Educational Psychology Review.

[B66-behavsci-15-01242] Wrench A., Garrett R., King S. (2013). Guessing where the goal posts are: Managing health and well-being during the transition to university studies. Journal of Youth Studies.

[B67-behavsci-15-01242] Xu J. (2022). More than minutes: A person-centered approach to homework time, homework time management, and homework procrastination. Contemporary Educational Psychology.

[B68-behavsci-15-01242] Yan Z., Lao H., Panadero E., Fernández-Castilla B., Yang L., Yang M. (2022). Effects of self-assessment and peer-assessment interventions on academic performance: A meta-analysis. Educational Research Review.

[B69-behavsci-15-01242] Yan Z., Wang X., Boud D., Lao H. (2023). The effect of self-assessment on academic performance and the role of explicitness: A meta-analysis. Assessment & Evaluation in Higher Education.

[B70-behavsci-15-01242] Zou H., Hastie T. (2005). Regularization and variable selection via the elastic net. Journal of the Royal Statistical Society: Series B (Statistical Methodology).

